# Mitigating an undesirable immune response of inherent susceptibility to cutaneous leishmaniosis in a mouse model: the role of the pathoantigenic HISA70 DNA vaccine

**DOI:** 10.1186/1297-9716-43-59

**Published:** 2012-08-09

**Authors:** Gustavo Domínguez-Bernal, Pilar Horcajo, José A Orden, Ricardo De La Fuente, Aldara Herrero-Gil, Lara Ordóñez-Gutiérrez, Javier Carrión

**Affiliations:** 1 Department of Animal Health, Veterinary Faculty, Complutense University of Madrid, 28040, Madrid, Spain; 2 “Severo Ochoa” Molecular Biology Centre CSIC-UAM, 28049, Madrid, Spain

## Abstract

*Leishmania major* is the major cause of cutaneous leishmaniosis (CL) outside of the Americas. In the present study we have cloned six *Leishmania* genes (H2A, H2B, H3, H4, A2 and HSP70) into the eukaryotic expression vector pCMVβ-m2a, resulting in pCMV-HISA70m2A, which encodes all six pathoantigenic proteins as a single polyprotein. This expression plasmid has been evaluated as a novel vaccine candidate in the BALB/c mouse model of CL. The DNA vaccine shifted the immune response normally induced by *L. major* infection away from a Th2-specific pathway to one of basal susceptibility. Immunization with pCMV-HISA70m2A dramatically reduced footpad lesions and lymph node parasite burdens relative to infected control mice. Complete absence of visceral parasite burden was observed in all 12 immunized animals but not in any of the 24 control mice. Moreover, vaccinated mice produced large amounts of IFN-γ, IL-17 and NO at 7 weeks post-infection (pi), and they showed lower arginase activity at the site of infection, lower IL-4 production and a weaker humoral immune response than infected control mice. Taken together, these results demonstrate the ability of the HISA70 vaccine to shift the murine immune response to *L. major* infection away from an undesirable, Th2-specific pathway to a less susceptible-like pathway involving Th1 and Th17 cytokine profiles.

## Introduction

The leishmaniases are a group of diseases caused by infection by several species of the intracellular protozoan parasites of the genus *Leishmania,* which are transmitted by the bite of an infected female phlebotomine sandfly. Clinical manifestations are divided into three broad categories, ranging from localized skin ulcers at the site of the sandfly bite (CL), to disfigurements (mucocutaneous leishmaniosis, MCL) and a potentially fatal disease of the viscera (visceral leishmaniosis, VL). These vector-borne diseases are a global public health problem, affecting an estimated 12 million people around the world. In fact, 1.5 million new cases of CL and 0.5 million new cases of VL are reported in humans each year [1].

Despite their great importance, the leishmaniases are among the most neglected tropical diseases (NTD) in the developing world [2]. This is mainly due to their strong association with poverty: leishmaniases have not become a very profitable market for the pharmaceutical sector. However, in recent years, these NTD have come to affect not only poor countries but also developed countries as a result of poor sanitary conditions as well as migration and travel from *Leishmania-*endemic areas [3-5]. In addition, climate change and other environmental changes have the potential to expand the geographical distribution of phlebotomine sandfly species [6]. Current control programs have focused their attention on vector and reservoir host control measures or mass drug distribution. Unfortunately, despite considerable progress, no vaccines are available to control any form of human leishmaniosis [7,8].

CL is caused by *Leishmania tropica* and *L. major* in Old World countries, and by *L. mexicana* and *L. amazonensis* in Central and South America. In this disease, infection of mammalian host phagocytes results in either subclinical infection or subacute to chronic disease characterized by lesions and scarring on exposed areas of the skin. Consequently, although the cutaneous form is not a lethal disease, people living with CL face significant social stigma. In addition, failure to treat patients with CL can give *Leishmania* parasites time to leave the skin lesions and invade the bloodstream to spread systemically in the host [9]. Moreover, depending on the species of *Leishmania* involved, cutaneous disease can progress to destructive MCL. Therefore, local or systemic treatment is important for shortening the disease duration, managing lesions, especially in the face, improving the cosmetic aspects of scarring, and avoiding the enormous social stigma [10,11]. Treatments with antimonial compounds and amphotericin B show variable efficacy, and they are also toxic and expensive [12]. Clearly, alternative treatment strategies and new prophylactic vaccines are needed.

Genetic vaccination is one option to efficiently prime specific, Th1 mediated host resistance against intracellular pathogens [13]. The efficacy of DNA vaccines against experimental leishmaniosis has recently been reviewed [14]. *Leishmania* is an intracellular parasite of mammalian phagocytic cells, such as macrophages and dendritic cells (DC). The outcome of the infection depends on the type of host immune response elicited. These phagocytic cells can control *Leishmania* infection when a Type 1 T helper (Th1) response is mounted, leading to the induction of inducible nitric oxide synthase (iNOS) and NO production, which is the main *Leishmania* killer molecule in the murine system [15]. However, *Leishmania* can develop several immune evasion tricks to persist in mammalian phagocytic cells. Cytokines released by Type 2 T helper (Th2) cells increase host cell arginase activity, producing polyamines that the pathogen uses for survival [16]. Therefore, the relative strength of the Th1 and Th2 responses remains the governing principle in *L. major* immunity. Insights into this theory of Th1/Th2 balance have come from studies in BALB/c mice, which show T-cell-mediated susceptibility to *L. major* infection. In this mouse model, development and progression of the disease requires sustained production of IL-4 by Th2 cells, whereas the Th1 response mediated by IL-12, IFN-γ, and TNF-α is associated with lesion resolution and control of parasite spread [11,17].

Recent developments are being used to investigate parasite virulence factors, elucidate immune regulatory mechanisms and contribute to the development of novel therapeutics and vaccines for the leishmaniases [18,19]. Recognition of *Leishmania* antigens by sera from patients and dogs suffering leishmaniosis is one of the methods most commonly used for diagnosis and identification of vaccine candidates against leishmaniosis [20-25]. Over the last decade, analysis of a *Leishmania* cDNA library and its successive fractionation into smaller libraries has resulted in the identification of novel protective antigens [26,27]. The sequence of events during *Leishmania* infection and the relevance of two distinct sets of parasite molecules have recently been reviewed [28]. Inside the susceptible mammalian host, *Leishmania* parasites produce surface, secreted and excreted antigens that help establish infection by preventing premature damage in both parasite and host cell. Later, intracellular parasite molecules are exposed to the host immune system. These intracellular antigens are considered “pathoantigenic” molecules, since they elicit immunological responses that contribute to disease pathology [29,30].

These pathoantigens were identified by Western blot analysis and/or immunoscreening of *Leishmania* expression libraries using patient sera [29]. They have attracted considerable attention because of two remarkable properties. First, they are recognized specifically by antibodies that do not cross-react with the host counterparts. This specificity, which makes pathoantigens a valuable tool in diagnosis, is due to the fact that their antigenic determinants lie in divergent regions of the sequence, as has been shown for *Leishmania* pathoantigens, namely nucleosome-forming histones (H2A, H2B, H3 and H4) [21,31], the amastigote-specific A2 protein [32], and the 70-kDa heat shock protein (HSP70) [20,24,25,33]. Second, immunization studies show that some of these pathoantigens can decrease the pathology-inducing Th2 immune response and increase a therapeutically more appropriate Th1-biased parasite-specific immune response [34-36].

The main goal of our project is to develop a pathoantigenic vaccine able to provide cross-protection against multiple *Leishmania* species. In order to design an improved vaccine against CL, we have cloned the full-length coding sequences of six *Leishmania* pathoantigenic genes (H2A, H2B, H3, H4, A2 and HSP70) into a mammalian expression vector that expresses the resulting polyprotein, called HISA70, in mammalian cells. The resulting plasmid, named pCMV-HISA70m2A, was used to immunize BALB/c mice against virulent *L. major* challenge.

## Materials and methods

### Vaccine preparation

**Figure 1:**
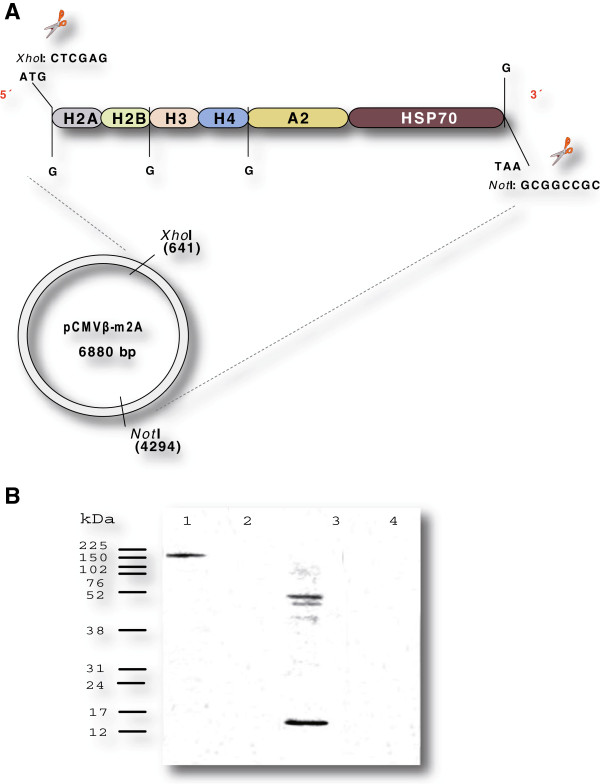
**Construction and testing of the plasmid DNA vaccine.** (**A**) Construction of pCMV-HISA70m2A. At the positions marked “G”, four tandem repeats of GGC, encoding four Gly residues, were inserted to improve structural flexibility of the HISA70 protein. (**B**) Analysis of HISA70 expression in CHO-K1 cells after transfection. Lane 1, cells transfected with pCMV-HISA70m2A, 148 kDa; lane 2, cells transfected with empty vector (pCMVβ-m2A); lane 3, nuclear extracts from *L. major* promastigotes, 13 kDa; lane 4, untransfected CHO-K1 cells. Protein expression was detected using specific anti-H2A sera from dogs with leishmaniosis. Proteins were separated on a 10% SDS-PAGE gel. The positions of molecular mass markers are indicated on the left.

DNA sequences of *Leishmania* genes were retrieved from the GeneDB and GenBank databases as follows: H2A (Lin J21.V3.1160), H2B (Lin.J09.V3.1410), H3 (LinJ10.V3.0920), H4 (Lin J31.V3.3320), A2 (GenBank **S69693**) and HSP70 (GenBank **CAA69282.1**). Then mouse codon-optimized versions of the sequences were generated using the GeneOptimizer® expert software system (Geneart AG) as described elsewhere [37]. The optimized sequences were synthesized chemically by GeneArt [38] as a single coding region of 4101 bp, named HISA70m. This sequence encodes a polyprotein of 1367 amino acid residues comprising, from N- to C-terminus, H2A-H2B-H3-H4-A2-HSP70. To facilitate further plasmid construction, *Xho*I and *Not*I sites were inserted, respectively, upstream and downstream of the HISA70m fusion gene, and this expression cassette was cloned into the eukaryotic expression plasmid pCMVβ-m2A [39]. Design of the plasmid DNA vaccine is shown in Figure 1A. 

The expression plasmid pCMV-HISA70m2A and empty vector pCMVβ-m2A were purified using the EndoFree plasmid Mega kit (Qiagen, Hilden, Germany) according to the manufacturer's recommended protocol. The final pellets of endotoxin-free DNA plasmid were resuspended in sterile PBS and stored at −20°C until their use in immunization.

### Transfection of plasmid constructs and Western blotting

Expression of HISA70 was detected in mammalian cells by transiently transfecting pCMV-HISA70m2A into CHO-K1 cells using Lipofectamine LTX and PLUS Reagent (Invitrogen, San Diego, CA, USA) according to the manufacturer's instructions. Briefly, CHO-K1 cells were cultured at a density of 4 × 10^4^ cells per well in 24-well plates in F-12K Nutrient Mixture with Kaighn’s modification (Invitrogen) containing 10% FCS and antibiotics; cells were transfected when they reached 50–80% confluence. Transfected cells were incubated 24 h at 37°C with 5% CO_2_. Then the cells were harvested and lysed by addition of 200 μL per well of Laemmli’s buffer (Bio-Rad) containing β-mercaptoethanol. Lysates (15 μL) were resolved by SDS-PAGE. Protein bands were electrophoretically transferred to a PVDF membrane (GE Healthcare, Madrid, Spain), which was blocked for 1 h in blocking buffer (10% non-fat milk in PBS-Tween-20). To detect the antigen blotted on the membrane, a primary antibody against *L. infantum* H2A was added at an appropriate dilution (1:200) and incubated with the membrane. This antibody was purified from a pool of anti-histone-positive sera from dogs suffering from VL [40]. Subsequently the membrane was blotted with a horseradish peroxidase-conjugated rabbit anti-dog IgG secondary antibody (1:2000; Cultek, Nordic Immunological Laboratories, Madrid, Spain).

### Experimental animals, parasites and preparation of soluble antigen

Eight-week-old female BALB/c mice were purchased from Harlan Interfauna Ibérica (Barcelona, Spain). The animals were maintained under standard conditions approved by the Ethics Committee of Complutense University of Madrid. *L. major* (clone V1: MHOM/IL/80/Friedlin) was cultured at 26°C in Schneider’s medium (Sigma-Aldrich, Madrid, Spain). Soluble *Leishmania* antigen (SLA) was prepared from stationary cultures of *L. major* promastigotes as previously described [41].

### Vaccination and infection of mice

Two groups of mice (*n* = 6) were immunized subcutaneously (s.c.) in the right footpad with 100 μg (in a volume of 40 μL) of the mammalian expression vector pCMVβ-m2A alone or with pCMV-HISA70m2A on days −60, -45 and −30. In parallel, a group of control mice (*n* = 6) was inoculated with PBS alone using the same procedure. On day 0 all groups of mice were infected by s.c. injection in the left footpad with 5 × 10^3^ metacyclic *L. major* promastigotes in a volume of 30 μL. Metacyclic forms were previously isolated from stationary cultures by negative selection using peanut agglutinin (Vector Laboratories, Barcelona, Spain) [42]. The course of infection was monitored weekly by measuring footpad swelling with a caliper. Clinical signs of ulceration were also assessed. Mice were euthanized by cervical dislocation at 7 weeks post-infection (pi), because at this time the lesions from non-vaccinated groups were larger than 4 mm in diameter or showed signs of ulceration. Draining lymph nodes (DLN) and spleens were removed from the euthanized mice. The experiments were carried out twice in order to evaluate the reproducibility of the approach.

### Generation of bone marrow-derived murine DC

Bone marrow cells were obtained from the femurs and tibiae of naïve BALB/c mice and cultured in the presence of 20 ng/mL murine granulocyte macrophage colony-stimulating factor (GM-CSF; PeproTech, London, UK), as previously described [43]. On day 7, bone marrow-derived DC were plated at 1 × 10^6^ cells/mL in 6-well plates and primed in the presence or absence of SLA (50 μg/mL). DC were collected at 24 h after pulsing with SLA and used for in vitro stimulation of T cells as described below.

### In vitro cell culture for determination of cytokine response and nitric oxide/nitrite production

At sacrifice 7 weeks pi, popliteal-DLN were harvested and prepared as single-cell suspensions as follows. DLN cells were washed, resuspended at a final concentration of 2 × 10^6^/mL in complete medium (DMEM supplemented with 10% heat-inactivated FCS, 2 mM L-glutamine, 100 U/mL penicillin, and 100 μg/mL streptomycin), and plated at 1 mL/well in 24-well plates containing bone marrow-derived DC (5 T cells : 1 DC) that had been left unstimulated or pulsed with SLA as described above. Cells were co-cultured for 96 h at 37°C and 5% CO_2_, and the culture supernatant was collected and stored at −20°C. Production of antigen-specific IFN-γ (Diaclone, Besançon, France), IL-4 (eBioscience, Barcelona, Spain) and IL-17 (R&D Systems, Madrid, Spain) was determined by ELISA according to the manufacturers’ suggested protocols. Levels of nitrite, which is a byproduct of nitric oxide production, were measured in culture supernatants using the Griess assay as previously described [44]. Briefly, 100 μL of culture supernatants were mixed with an equal volume of Griess reagent (Sigma-Aldrich) and incubated at room temperature for 10 min. Absorbance was then measured at 540 nm.

### Arginase activity assay

Arginase activity at the site of lesions was determined ex vivo using 5–10 mL of footpad homogenate as previously described [45,46]. Briefly, *L. major*-infected footpads were rinsed in ethanol and homogenized by hand in PBS containing 100 U/mL penicillin and 100 μg/mL streptomycin. The homogenate was first centrifuged at low speed (50 *g*) for 5 min to remove large tissue debris, and the supernatant was then centrifuged again in PBS at high speed (1811 *g*) for 15 min to pellet phagocytic cells and amastigotes. Both pellets were incubated for 30 min in 2 mL of lysis buffer (0.1 M Tris–HCl, 300 μM NaCl, 1 μM PMSF, 1% Triton X-100) and the lysate was assayed for arginase activity as previously described [47,48]. One unit of enzyme activity is defined as the amount of enzyme that catalyzes the formation of 1 mmol of urea/min.

### Estimation of parasite burden

Parasite burden in the ipsilateral popliteal-DLN and spleen was determined at 7 weeks pi by limiting dilution culture [49]. Briefly, the organs were harvested and a suspension was prepared by grinding the tissue in 1 mL of Schneider’s medium (Sigma-Aldrich) containing 20% FCS in the presence of streptomycin and penicillin. Four-fold serial dilutions of the homogenized tissue suspensions were plated in a 96-well culture plate and incubated at 26°C for 10 days. Wells were examined for viable promastigotes. The reciprocal of the highest dilution factor that was positive for promastigotes was considered to be the number of *Leishmania* parasites per mg of tissue.

### Humoral immune response

*Leishmania*-specific antibodies were quantified by ELISA. Standard plates were coated overnight at 4°C with 100 μL of SLA (2 μg/mL) diluted in PBS. Afterwards, wells were washed with PBS supplemented with 0.05% (v/v) Tween-20 and blocked with 2% (w/v) BSA in PBS. Sera were serially diluted in order to determine the titer, which was defined as the inverse of the highest serum dilution factor giving an absorbance > 0.2. Secondary antibodies were peroxidase-conjugated goat anti-mouse IgG, IgG1 and IgG2a (1:500, SouthernBiotech, Madrid, Spain). After washing and addition of peroxidase substrate (ABTS, Roche Diagnostics, Madrid, Spain), sample absorbance was measured at 405 nm.

### Statistical analysis

Since statistical analysis showed that measurements followed the standard normal distribution, Student’s *t*-test was used to evaluate the significance of differences between means in the control and experimental groups; means were the average from two independent experiments. Differences were considered significant when *P* < 0.05. Statistical analyses were performed using SigmaPlot software (version 12.2, Systat Software).

## Results

### HISA70 protein can be expressed in CHO-K1 cells

Expression of recombinant proteins by host mammalian cells transfected with plasmid DNA is an essential requirement for stimulating the host immune system. Expression of HISA70 in CHO-K1 cells transfected with pCMV-HISA70m2A was easily detected using an antibody against *L. infantum* H2A (Figure 1B).

### Vaccination with pCMV-HISA70m2A confers protection against *L. major* infection in BALB/c mice

**Figure 2:**
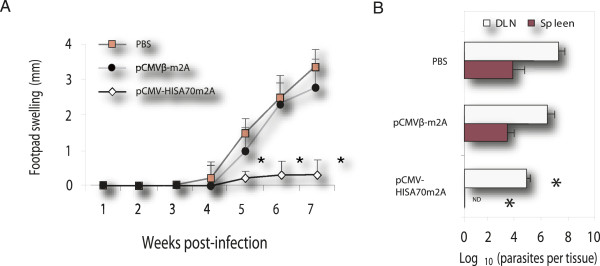
**Increased footpad swelling correlated with high parasite burden in control mice at 7 weeks pi.** (**A**) Course of footpad lesion development in BALB/c mice infected with *L. major*. (**B**) Parasite burden in the popliteal-DLN and spleen were determined at 7 weeks pi by limiting dilution (see Materials and Methods). ND: parasites not detected. Asterisks indicate *P* < 0.05 with respect to both groups of control mice. Results are the mean and SD (bars) in groups of mice (*n* = 6) from two independent experiments.

As expected, immunization of mice with pCMVβ-m2A alone had no effect on lesion development during the course of infection. These mice succumbed to progressive disease. Indeed, the size of footpad swellings (Figure 2A) and parasite burdens (Figure 2B) in the popliteal-DLN and spleen of these mice were not significantly different from those of control mice (PBS) at 7 weeks pi. In contrast, mice immunized with pCMV-HISA70m2A did not develop lesions at the site of infection or, in some cases, they developed significantly smaller footpad lesions up to 5 weeks after challenge with *L. major,* with no signs of ulcer formation or necrotic tissue. Moreover, the numbers of parasites in popliteal-DLN were significantly lower in mice immunized with pCMV-HISA70m2A than in control mice (PBS or pCMVβ-m2A alone), and immunized mice did not show the visceralization observed in the control animals (Figure 2B).

### Vaccination with pCMV-HISA70m2A redirects the *L. major*-specific Th2 immune response to a less susceptible-like phenotype

In order to elucidate the type of immune responses elicited by *L. major* infection, we assessed the production of IFN-γ, IL-17 and IL-4 in the popliteal-DLN after stimulation with SLA. As shown in Table 1, DLN cells of mice vaccinated with pCMV-HISA70m2A produced significantly lower levels of specific IL-4 than those of control mice, but they also produced significantly larger amounts of IFN-γ and IL-17 at 7 weeks pi. In contrast, DLN cells of mice immunized with pCMVβ-m2A alone produced substantial amounts of IL-4, as well as levels of IFN-γ and IL-17 similar to those of non-vaccinated mice.

**Table 1:** Cytokine production at 7 weeks pi in mice vaccinated with pCMV-HISA70m2A and in control mice

**pg/mL**			
	**IFN-γ**	**IL- 17**	**IL- 4**
**PBS**	600 ± 100	932 ± 50	491 ± 115
**pCMVβm2A**	650 ± 100	1035 ± 50	380 ± 80
**pCMV-HISA70m2A**	1102 ± 150 (*)	1300 ± 50 (*)	212 ± 35 (*)

Data are presented as means ± SD and are representative of two independent experiments with similar results. Asterisks indicate *P* < 0.05 with respect to both groups of control mice.

### Competition between iNOS and arginase enzymes in phagocytic cells contribute to the outcome of *L. major* infection

**Figure 3:**
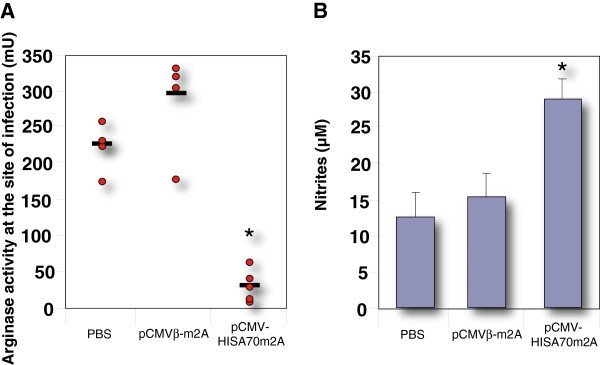
**Mice vaccinated with pCMV-HISA70m2A showed low tissue arginase activity at the site of infection as well as impaired nitrite production.** (**A**) BALB/c mice were sacrificed at 7 weeks pi and arginase activity was determined in infected footpad homogenates. (**B**) In the same experiments, nitrite levels were assayed in medium from cultures of DLN draining the infection site (see Materials and Methods). Results shown are means and SD (bars) for groups of mice (*n* = 6) from two independent experiments. Asterisks indicate *P < 0.05 with respect to both groups of control mice.

Our data showed that arginase activity at the site of infection was significantly lower in vaccinated mice than in control animals (Figure 3A). We also measured iNOS activity indirectly by assaying nitrite production after stimulating DLN cells with SLA-pulsed DC. We found significantly higher nitrite levels in cells of mice vaccinated with pCMV-HISA70m2A than in those of non-vaccinated mice (Figure 3B). Conversely, no significant difference between control and pCMVβ-m2A immunized mice was found at 7 weeks pi.

### Characterization of the specific humoral response in immunized mice

**Figure 4:**
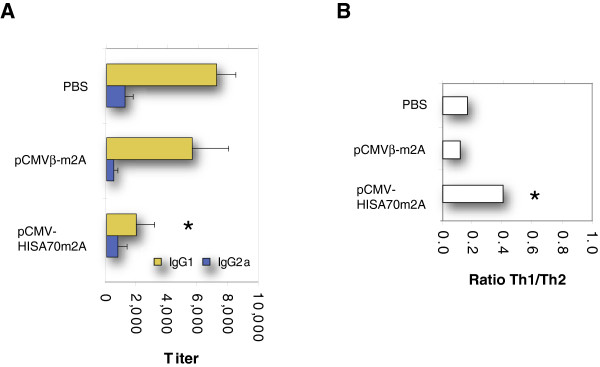
**Anti-SLA IgG1 antibodies predominated over IgG2a in non-protected mice.** (**A**) Sera were collected at 7 weeks pi from mice vaccinated with pCMV-HISA70m2A and control mice treated with PBS alone or with empty vector. Titers of anti-SLA IgG1 and IgG2a antibodies were determined by ELISA. (**B**) *Leishmania*-specific IgG2a : IgG1 ratio. Asterisks indicate *P* < 0.05 with respect to both groups of control mice (PBS only, empty vector only). Results are the mean and SD (bars) for groups of mice (*n* = 6) from two independent experiments.

Previous studies in the CL mouse model have reported that the relative production of immunoglobulin isotypes correlates with the relative strength of induction of Th1- and Th2-biased immune responses [50,51]. To characterize the humoral immune response to *L. major* infection, we measured levels of IgG1 and IgG2a isotypes in sera of control and immunized mice at 7 weeks pi (Figure 4A). This analysis revealed that levels of *Leishmania*-specific IgG1 antibodies were significantly lower in mice immunized with pCMV-HISA70m2A than in control mice. Consequently, the mean Th1/Th2 humoral ratio after infection was significantly higher in mice vaccinated with pCMV-HISA70m2A than in the two control groups (Figure 4B). 

## Discussion

Attempts to develop immunization protocols to protect against *Leishmania* infection have met with mixed success. A series of studies have documented resistance to infection after immunization with *Leishmania* pathoantigens. Table 2 summarizes data from preclinical studies of potential vaccines in animal models; these vaccines involve one or more of the six intracellular pathoantigens used to design the HISA70 vaccine in the present work (H2A, H2B, H3, H4, A2 and HSP70).

**Table 2:** **Vaccines based on six *Leishmania *pathoantigens (H2A, H2B, H3, H4, A2 and HSP70)**

**Antigen**	**Targeted disease**	**Animal model**	**Delivery system**	**Result**	**Reference**
H2A/H2B/H3/H4	CL	BALB/c	DNA vaccine	Protection	[34]
N-terminal part-H2B	CL	BALB/c	Protein + CpG	Protection	[52]
H2A/H2B/H3/H4	CL	BALB/c	Ag-loaded DCs	Long-lasting protection	[53]
H2A/H3, H2A/H4, H2B/H3, H2B/H4	CL	BALB/c	DNA vaccine	Partial protection	[40]
H2A/H2B, H3/H4	CL	BALB/c	DNA vaccine	Partial protection	[54]
H1/H2A/H2B/H3/H4 + 5 more Ags	VL	Dog	DNA vaccine	Partial protection (DLN)	[55]
H2A/H2B/H3/H4	VL	BALB/c	DNA vaccine	No protection	[56]
H2A/H2B/H3/H4	VL	BALB/c	Ag-loaded DCs	Partial protection	[56]
A2	CL	BALB/c	Protein + IL-12	Partial protection	[57]
A2	CL/VL	BALB/c	DNA vaccine	Partial protection	[58]
A2	VL	BALB/c	DNA vaccine	Partial protection (liver)	[59]
A2	VL	BALB/c	Protein + *P. acnes*	Protection (liver)	[60]
A2	VL	Dog	Protein + saponine	Partial protection	[61]
A2	VL	BALB/c	A2-Adenovirus	Protection	[62]
A2	VL	BALB/c	*L. tarentolae*	Protection	[63]
A2	VL	BALB/c	Live bacteria	Partial protection	[64]
HSP70	CL	BALB/c	Primeboost	No protection	[65]
HSP70 + HSP83	VL	BALB/c	Protein ± ALD/MPLA	Protection (liver)	[66]
GP63 + HSP70	VL	BALB/c	DNA vaccine	Protection	[67]
GP63 + HSP70	VL	BALB/c	Protein	Protection	[68]

Immunization studies in mice using various combinations of *Leishmania* nucleosomal histones, either encoded in DNA plasmids or pulsed into dendritic cells, have demonstrated different levels of protection against CL [40,53] and VL [56]. Unfortunately, only partial reduction of parasite burden in lymph nodes has been reported against canine VL [55]. Recent studies have suggested that the presence or absence of immunodominant epitopes on histones strongly influences *Leishmania* parasite persistence, and that Treg cells mediate this effect, suggesting the complexity of the immunization process [52,54].

The immunogenic properties of *Leishmania* A2 antigen have been extensively described [69-73]. Several vaccine approaches using the A2 antigen have conferred different levels of protection against murine VL [58-60,62-64]. Vaccination of dogs using A2 confers partial protection to canine VL [61], which is significant because dogs are the main domestic reservoir of viscerotropic species of *Leishmania*. Furthermore, immunizing mice with a combination of A2 protein and IL-12 or with an expression vector encoding A2 protected them against challenge with *L. amazonensis*, the causative agent of CL in Latin America [57,58]. Curiously, no studies have analyzed the ability of A2 antigen vaccines to protect against *L. major* infection, which is responsible for CL in humans in the Old World. The most likely reason is that the *L. donovani* A2 protein coding sequence, which is the only one tested so far in vaccines, is not present in the genome of all *Leishmania* species and appears to be absent from *L. major* and *L. tropica*[74].

HSP are abundant intracellular proteins present in all cells. Since their discovery, HSP have been assigned various functions, such as folding, assembly, protein transport and thermotolerance. After exposure to various forms of shock, such as exposure to high temperatures (“heat shock”), toxins, oxidative stress, and glucose deprivation, HSP levels can rise to protect cellular proteins against denaturation [75]. HSP also bind antigenic peptides and interact with antigen-presenting cells. Microbial HSP have been implicated in the induction of the innate and adaptive arms of the immune response [76,77]. Interestingly, when HSP bind to antigenic peptides, the resulting complexes can prime T cell immunity specifically against the bound peptides, but not against HSP themselves [78]. Indeed, vaccines based on HSP-peptide complexes have recently been considered for immunotherapy against cancers and infectious diseases [79].

Among HSP, HSP70 stands out because its complexes with antigenic peptides can generate a peptide-specific cellular immune response four orders of magnitude more efficient than the peptide alone [77]. In the same way, *Leishmania* GP63 antigen administered without adjuvant elicited partial protection in mice, but when it was coadministered with HSP70, it conferred protection against VL [67,68]. Furthermore, a cocktail of HSP70 and HSP83 seems to reduce hepatic parasite burden against murine VL [66]. In contrast, other authors failed to demonstrate protection after immunization with HSP70 alone in a murine CL model [65]. These findings are consistent with the role of HSP70 as an adjuvant in antigen presentation, and they suggest that HSP70 must be bound to antigen to induce an immune response. This makes sense, because HSP are self-antigens, so they should not elicit immune responses against themselves [78,80]. Thus, vaccines based on complexes of HSP and microbial peptides show promise as safe and effective therapies against intracellular pathogens.

Given the immunogenicity of all six intracellular *Leishmania* pathoantigens, we developed a DNA vaccine that encodes all six proteins as a single polyprotein (HISA70). In the present study we found that DNA immunization of BALB/c mice with pCMV-HISA70m2A induced protection against *L. major* infection. Normally, after *Leishmania* invasion in BALB/c mice, infected phagocytic cells are stimulated by a Th2-type signal, cytokine IL-4, to express arginase [81]. This inducible enzyme initiates L-arginine degradation, leading to the synthesis of polyamines, which are necessary for *Leishmania* parasite growth in infected cells [16]. In the present study, popliteal-DLN of vaccinated mice showed low levels of *Leishmania*-specific IL-4 at 7 weeks pi, which led to significantly lower arginase activity at the site of the lesion in mice vaccinated with pCMV-HISA70m2A than in control mice. This in turn allowed the development of a protective anti-*Leishmania* Th1 response mediated by NO-dependent IFN-γ production. This was in accordance with previous findings that phagocytic cells can control *Leishmania* infection when a Th1 response is mounted and high levels of NO are produced [15]. NO is the most important *Leishmania* killer molecule in the mouse, and levels of nitrite, a byproduct of NO synthesis, were significantly higher in the vaccinated mouse than in the control animals.

In addition to nitrite, our mice vaccinated with pCMV-HISA70m2A expressed significantly higher levels of specific IL-17 than did the control mice. Our finding of elevated IL-17 levels in vaccinated mice is consistent with previous studies in which parasite-specific cellular responses shifted towards Th1 and Th17 mechanisms of protective immunity in both VL and CL murine models [82,83]. IL-17 favors the production of chemokines that mediate the recruitment of neutrophils as well as Th1 cells [84,85]. Th17 cells secrete IL-17, IL-21, and IL-22, which exert strong proinflammatory effects that play a crucial role in fighting Gram-negative bacteria, fungi, and some protozoa [86]. Indeed, IL-17 mediates control of parasitemia in mice infected with *Trypanosoma congolense*[87].

We found that the HISA70 vaccine did not enhance the humoral response in mice. In addition, we assessed the relative production of IgG2a and IgG1 isotypes, since these have been widely used as markers, respectively, of the induction of Th1- and Th2-type immune responses [51]. Vaccinated mice showed a lower titer of the anti-SLA IgG1 isotype than did control mice. In contrast, SLA-specific IgG1 antibodies predominated in control mice. Thus, vaccination increased the ratio of IgG2a/IgG1 in protected mice.

In conclusion, vaccination with pCMV-HISA70m2A successfully promoted Th1- and Th17-type immune responses, thereby mitigating the Th2 response caused by *L. major* infection and creating a resistant-like phenotype. As a result, vaccinated mice developed much fewer and slower-growing lesions than did control mice, and showed a lower parasite burden at the site of infection, with no visceralization. These findings suggest the usefulness of HISA70 for designing vaccines with improved protective efficacy against murine CL.

## Competing interests

The authors declare that they have no competing interests.

## Authors’ contributions

GDB participated in the design and coordination of the study, and helped to draft the manuscript. PH, JAO, RDF and AHG participated in its design and the discussion section. LOG carried out the detection of HISA70 expression in mammalian cells and participated in the discussion section of this paper. JC conceived the study, carried out the experiments and wrote the paper. All authors read and approved the final manuscript.

## Authors’ information

Complutense University of Madrid has filed a patent on the HISA70 as a DNA vaccine against leishmaniosis.
